# Construction of an immune-related ceRNA network to screen for potential diagnostic markers for autism spectrum disorder

**DOI:** 10.3389/fgene.2022.1025813

**Published:** 2022-11-17

**Authors:** Jing-Jing Sun, Bo Chen, Tao Yu

**Affiliations:** ^1^ Department of Pediatrics, Shengjing Hospital of China Medical University, Shenyang, Liaoning, China; ^2^ Disabled Service Center of Liaoning Province, Shenyang, Liaoning, China

**Keywords:** autism spectrum disorder (ASD), competitive endogenous RNA, immune cell, bioinformatics analysis, diagnostic biomarkers

## Abstract

**Purpose:** The diagnosis of autism spectrum disorder (ASD) is reliant on evaluation of patients’ behavior. We screened the potential diagnostic and therapeutic targets of ASD through bioinformatics analysis.

**Methods:** Four ASD-related datasets were downloaded from the Gene Expression Omnibus database. The “limma” package was employed to analyze differentially expressed messenger (m)RNAs, long non-coding (lnc)RNAs, and micro (mi)RNAs between ASD patients and healthy volunteers (HVs). We constructed a competing endogenous-RNA (ceRNA) network. Enrichment analyses of key genes were undertaken using the Gene Ontology database and Kyoto Encyclopedia of Genes and Genomes database. The ImmucellAI database was used to analyze differences in immune-cell infiltration (ICI) in ASD and HV samples. Synthetic analyses of the ceRNA network and ICI was done to obtain a diagnostic model using LASSO regression analysis. Analyses of receiver operating characteristic (ROC) curves were done for model verification.

**Results:** The ceRNA network comprised 49 lncRNAs, 30 miRNAs, and 236 mRNAs. mRNAs were associated with 41 cellular components, 208 biological processes, 39 molecular functions, and 35 regulatory signaling pathways. Significant differences in the abundance of 10 immune-cell species between ASD patients and HVs were noted. Using the ceRNA network and ICI results, we constructed a diagnostic model comprising five immune cell-associated genes: adenosine triphosphate-binding cassette transporter A1 (*ABCA1*), DiGeorge syndrome critical region 2 (*DGCR2*), glucose-fructose oxidoreductase structural domain gene 1 (*GFOD1*), glutaredoxin (*GLRX*), and SEC16 homolog A (*SEC16A*). The diagnostic performance of our model was revealed by an area under the ROC curve of 0.923. Model verification was done using the validation dataset and serum samples of patients.

**Conclusion:** *ABCA1, DGCR2, GFOD1, GLRX*, and *SEC16A* could be diagnostic biomarkers and therapeutic targets for ASD.

## Introduction

Autism spectrum disorder (ASD) is a group of heterogeneous neurodevelopmental disabilities characterized by an early onset of impaired social function, abnormal stenosis, and repetitive behaviors and interests (Lord et al., 2018). Worldwide ASD prevalence is approximately 0.8–1.7%, and the ratio of ASD in males:females varies between 2:1 and 4:1 (Mattila et al., 2011; Russell et al., 2014). ASD etiology is incompletely understood, but genetic, environmental, and immune factors may play a part in its pathogenesis (McAllister, 2017; Vorstman et al., 2017). Efficacious drugs for the core symptoms of ASD are lacking. Some antiepileptic and psychotropic drugs have been approved for treating the epilepsy and mood abnormalities associated with ASD (Berry-Kravis et al., 2018).

Behavioral and educational interventions remain the most popular treatment measures for ASD. Early behavioral interventions improve the core symptoms of ASD patients, as well as their verbal and cognitive ability and social adaptability (Lai et al., 2014; Lord et al., 2018). Several genes have been associated with ASD, and many metabolites (e.g., amino acids, organic acids, phospholipids, and purines) show significantly different expression in ASD patients (Vorstman et al., 2017; Likhitweerawong et al., 2021). However, due to heterogeneity among ASD patients, the diagnosis of ASD is reliant on evaluation of the patient’s behavior. Therefore, identifying the characteristic molecular markers may contribute to understanding of the pathophysiological mechanism of ASD, thereby increasing the chance of a correct diagnosis and improvement in therapeutic interventions.

A microRNA (miRNA) is a non-coding RNA with a length of ∼22 nucleotides. miRNAs can inhibit the translation of target messenger (m)RNAs by binding to partially complementary sequences within them (Correia de Sousa et al., 2019). The sequence fragments on the target mRNA that can bind to the miRNA are called “miRNA response elements” (MREs) (Salmena et al., 2011). In 2011, Salmena et al. first proposed the concept of “competitive endogenous RNAs” (ceRNAs), which are different long non-coding-RNAs (lncRNAs), mRNAs, circular RNAs (circRNAs), or pseudogenes with identical MREs that can bind to the same miRNA competitively and interact with each other indirectly. Thus, these RNAs compete with and regulate each other’s expression, with miRNA as the core, forming a complex transcriptional regulatory network (Salmena et al., 2011). For example, as the main type of ceRNA, in a lncRNA–miRNA–mRNA network, lncRNA and mRNA interact indirectly by sharing the same target miRNA. In short, lncRNA can downregulate miRNA expression, whereas miRNA can downregulate mRNA expression; thus, lncRNA can upregulate mRNA expression indirectly. Conversely, mRNA can also increase lncRNA expression. This action helps to coordinate the proliferation, differentiation, and apoptosis of cells (Schmitz et al., 2016). Multiple studies have shown that cancer, ischemic stroke, Parkinson’s disease, and other diseases are related to an abnormal ceRNA network comprising lncRNA–miRNA–mRNA (Long et al., 2019; Zhang et al., 2020; Berti et al., 2021; Li et al., 2021). However, the specific role of a ceRNA network in ASD has not been elucidated.

**FIGURE 1:**
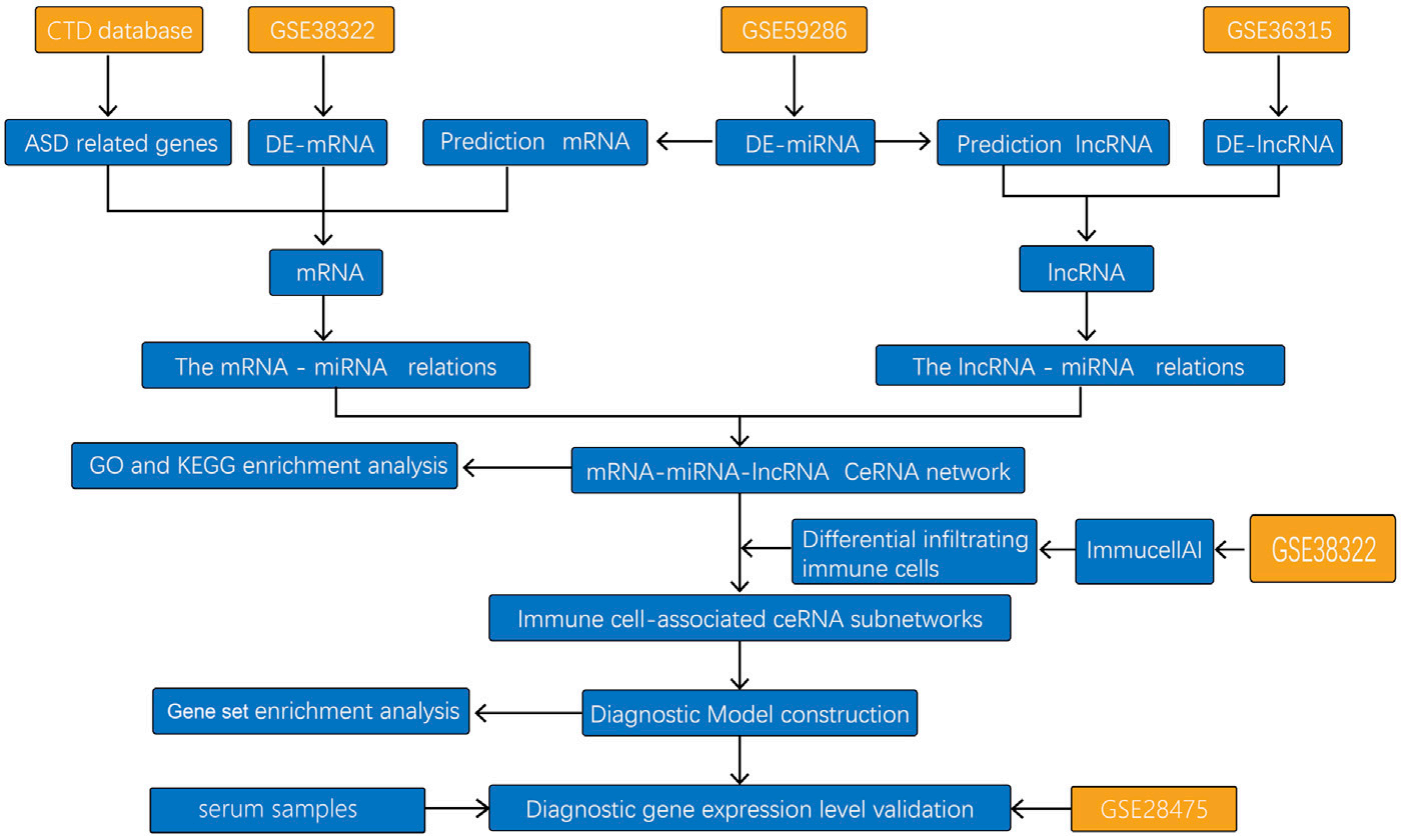
Study flowchart.

We aimed to screen the potential diagnostic markers and therapeutic targets of ASD from the perspective of a ceRNA network. We downloaded ASD-related datasets from the Gene Expression Omnibus (GEO) database to screen differentially expressed mRNAs, lncRNAs, and miRNAs in ASD patients to construct a ceRNA network. Based on analyses of differences in the infiltration of immune cells between samples from ASD patients and healthy volunteers (HVs), and combining analyses of the ceRNA network, diagnostic marker genes were obtained by least absolute shrinkage and selection operator (LASSO) regression analyses. Finally, we validated the diagnostic model through validation datasets and samples of peripheral blood from ASD patients (Figure 1).

## Materials and methods

### Data collection

By searching the GEO database (www.ncbi.nlm.nih.gov/geo/), 19 datasets were found pertaining to ASD (*Homo sapiens*): five brain-sample datasets, 11 blood-sample datasets, and three stem cell- or fibroblast-sample datasets. Considering the tissue differences in gene expression, we chose four brain datasets (GSE38322, GSE36315, GSE59286, and GSE28475) for analyses (GSE102741 was eliminated because it focused mainly on genes related to the histamine system). mRNA expression data were acquired from the GSE38322 dataset (Ginsberg et al., 2012), which contained brain transcriptional data of 18 ASD and 18 HVs samples used to detect DE-mRNAs. The lncRNA expression profile was downloaded from the GSE36315 dataset (Ziats and Rennert, 2013), including brain transcriptional data of four ASD and four HVs samples, which was used to screen DE-lncRNAs. miRNA high-throughput sequencing data acquired from the GSE59286 dataset, which contained brain transcriptional data of 25 HVs and 20 ASD samples, were used to select DEmiRNAs. The expression profile data were downloaded from the GSE28475 dataset (Chow et al., 2011), which contained brain transcriptional data of 61 HVs and 52 ASD samples and served as a validation set. The demographic and clinical characteristics of samples from the GSE38322, GSE36315, GSE59286, and GSE28475 datasets are shown in Table 1.

**TABLE 1:** Demographhic and clinical characteristics of samples from the datasets.

Variables	GSE38322	GSE36315	GSE59286	GSE28475
Total (N)	36	8	45	113
Control	18	4	25	61
ASD	18	4	20	52
Sample type	Occipital cortex/Cerebellum	Prefrontal cortex/cerebellum	Superior frontal gyrus	Superior frontal gyrus
Mean Age (range)	21 (1–60)	10 (4–16)	16 (1–62)	19 (2–56)
Gender (male/female)	No data	8/0	31/14	No data
Ethnicity	No data	Caucasian	No data	No data
Countries and regions	USA/Cleveland	USA/Bethesda	China/Shanghai	USA/La Jolla

### Gene screening

The mRNAs, lncRNAs, and miRNAs that showed different expression between ASD patients and HVs were screened in the corresponding datasets using “limma 3.5.1” within R (R Institute for Statistical Computing, Vienna, Austria). The screening thresholds were *p* < 0.05 and |log_2_ fold change (FC)| >0.5 to obtain genes with significantly different expression.

### Construction of a ceRNA network

The predicted target mRNA was obtained by predicting the target mRNA of differentially expressed miRNA in the GSE59286 dataset using the miRWalk database (http://mirwalk.umm.uni-heidelberg.de/). In the Comparative Toxicogenomics Database (CTD; http://ctdbase.org/), 24,785 genes related to ASD were retrieved using “Autism” as the keyword. At the intersection of predicted target mRNAs and ASD-related genes in the CTD, mRNAs that showed differential expression in the GSE38322 dataset were obtained using Venn (http://bioinformatics.psb.ugent.be/webtools Venn/). Subsequently, miRNA–mRNA pairs conforming to the regulation principles of miRNA and mRNA in the ceRNA network were obtained.

The LncBaseV3 database (https://diana.e-ce.uth.gr/lncbasev2/home/) was used to predict the target lncRNAs of differentially expressed miRNAs obtained in the GSE59286 dataset, and a threshold score >0.6 was set. Venn was employed to obtain the intersection of predicted lncRNAs and differentially expressed lncRNAs in the GSE36315 dataset. According to the functional mechanism of ceRNAs, differentially expressed miRNAs that had a regulatory relationship with the obtained lncRNA were acquired to construct miRNA–lncRNA pairs. Finally, according to the miRNA–mRNA pairs and miRNA–lncRNA pairs obtained, the lncRNA–miRNA–mRNA associations with the same miRNA as the core were screened to construct the ceRNA network, and Cytoscape (https://cytoscape.org/) was used to visualize the results.

### Infiltration of immune cells

Analyses of the infiltration of immune cells were undertaken on the brain-tissue samples of 18 HVs and 18 ASD patients from the GSE38322 dataset using the ImmucellAI database (http://bioinfo.life.hust.edu.cn/web/ImmuCellAI/). Then, Student’s *t*-tests were undertaken using “ggplot2” in R to analyze the different immune cells between samples from ASD patients and HVs. The correlation between significantly different immune cells and mRNAs in the ceRNA network was analyzed by the Spearman method using the “ggstatsplot” in R. The relationship between different immune cell–mRNA pairs was screened using a threshold of *p* < 0.05 and correlation coefficient |r| > 0.8.

### Screening and identification of diagnostic markers

Based on the obtained mRNAs associated with immune cells and their expression in each sample of the GSE38322 dataset, “glmnet” v4.0-2 in R (https://cran.r-project. org/web/packages/glmnet/index.html/) was used for the LASSO screening of diagnostic genes for ASD. The parameters were set as family = “binomial” and n fold = 20 (i.e., 20-fold cross-validation was undertaken to screen for diagnostic markers). The eigenvalue of each sample was calculated based on the regression coefficient of screened genes and expression of the genes in the GSE38322 dataset using the formula :

$${feature}_{sample}=\overset{n}{\underset{1}{\sum }}{Coef}_{i}*x_{i}$$

where Coef_i_ denotes the LASSO regression coefficient of the NO. i gene, xi denotes expression of the NO. x gene, and n denotes the number of diagnostic markers in the diagnostic model. The receiver operating characteristic (ROC) curve was plotted using “pROC” within R to assess the predictive efficacy of the diagnostic model and diagnostic markers for the disease.

### Enrichment analyses

Analyses of the enrichment of the function and signaling pathways of mRNAs in the ceRNA network were done using the Gene Ontology database (GO; http://geneontology.org/) and Kyoto Encyclopedia of Genes and Genomes (KEGG; https://www.genome.jp/kegg/) database, respectively, using “cluster Profiler” in R. The relevant biological processes, molecular functions, cellular components, and regulatory signaling pathways involved were obtained using a significance threshold of *p* < 0.05. Gene set enrichment analysis (GSEA) was done for each diagnostic marker gene using GSEA v4.0.3 (www.gsea-msigdb.org/gsea/index.jsp/). Enrichment analyses using GO and KEGG databases were conducted using GSEA for high and low expression of the diagnostic biomarker genes. An FDR q-value < 0.05 was considered to denote significant enrichment.

### Validation of expression of diagnostic genes

“ggplot2” in R was employed to undertake Student’s *t*-tests to measure expression of the diagnostic markers in samples from ASD patients and HVs in the GSE28475 dataset, and the results are presented as boxplots.

### Real-time reverse transcription-quantitative polymerase chain reaction of serum samples

This study was conducted in accordance with the Declaration of Helsinki 1964 and its later amendments. The study protocol was approved by the medical ethics committee of Shengjing Hospital of China Medical University (Liaoning, China). Written informed consent was obtained from all participants.

Blood samples from 10 ASD patients were obtained from the Liaoning Disability Service Center (Liaoning, China) and blood samples from 10 HVs were obtained from Shengjing Hospital of China Medical University between January 2022 to March 2022. Supplementary Table S1 shows specific information of these individuals.

We isolated the serum from whole blood. Then, we extracted the total RNA from serum using TRIzol™ Reagent (Ambion, Austin, TX, United States). Reverse transcription was undertaken using the SweScript RT I First Strand cDNA Synthesis Kit (Servicebio, Wuhan, China). PCRs were carried out using the 2× universal Blue SYBR Green qPCR Master Mix Kit (Servicebio) according to manufacturer instructions. The reaction conditions were 95°C for 1 min (initial denaturation), 95°C for 20 s (denaturation), 55°C for 60 s (annealing), and 72°C for 30 s (extension) and 40 cycles in total. Relative quantification was undertaken with the 2^−ΔΔCT^ method, and the gene for glyceraldehyde 3-phosphate dehydrogenase (GAPDH) was used as the internal control for normalization.

The primer sequences (forward and reverse, respectively) used were: 5′-CTG​GGA​AGG​TGG​TTG​TGT​T-3′ and 5′-GTG​GTT​GGT​GGC​TGT​GAT​A-3′ for GLRX; 5′-ACC​CGT​CCA​TCC​TAC​ATC-3′ and 5′-TCA​CCT​CGT​CTC​TCT​TCC​GTC-3′ for SEC16A; 5′-GCG​GGG​AGA​ACT​ACT​GGG​AT-3′ and 5′-GTG​GAG​AAG​GTG​GCG​AGA​GA-3′ for DGCR2; 5′-GAC​TGC​CTG​TAT​GCC​TTG​T-3′ and 5′-CCG​TCT​GGC​TGG​ACC​TCT​T-3′ for GFOD1; 5′-TCC​TCT​TTC​CCG​CAT​TAT​CT-3′ and 5′-GTC​CAT​TTC​TTG​GCT​GTT​CT-3′ for ABCA1; 5′-CCC​ATC​ACC​ATC​TTC​CAG​G-3′ and 5′-CAT​CAC​GCC​ACA​GTT​TCC​C-3′ for GAPDH.

### Statistical analyses

Statistical analyses were undertaken using R 3.6.2. The Student’s *t*-test was used to compare gene expression among serum samples, and *p* < 0.05 was considered significant.

## Results

### Differentially expressed lncRNAs, miRNAs, and mRNAs

**FIGURE 2:**
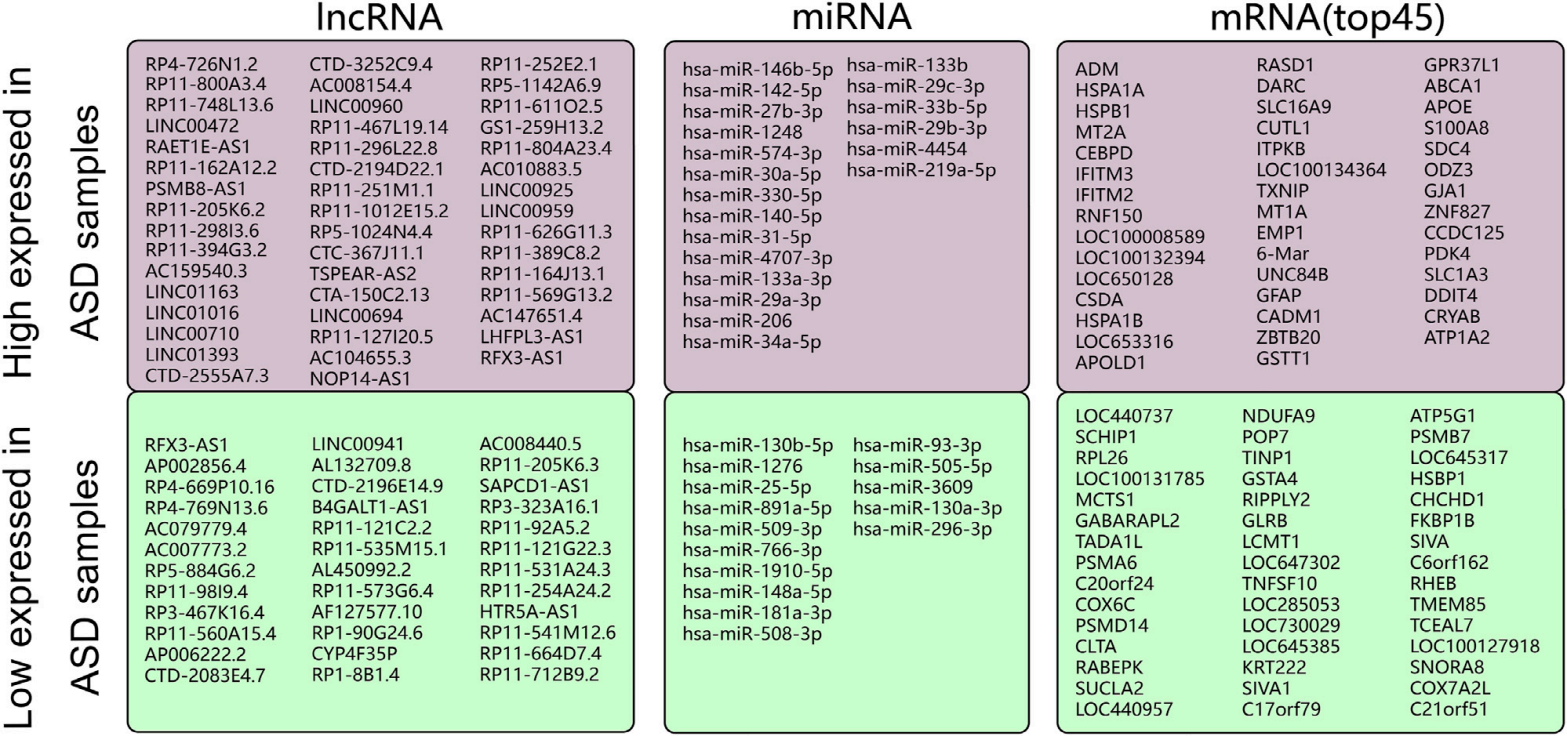
Differentially expressed lncRNAs, miRNAs, and mRNAs (top 45) between ASD patients and HVs in the dataset.

According to the screening conditions that we set (*p* < 0.05 and |log_2_ FC| >0.5), 662 differentially expressed mRNAs were obtained in the GSE38322 dataset, of which 295 had upregulated expression and 367 had downregulated expression in the samples from ASD patients and HVs, respectively (Figure 2, Supplementary Table S2). Furthermore, 83 differentially expressed lncRNAs and 35 differentially expressed miRNAs were obtained from the GSE36315 dataset and GSE59286 dataset, respectively, of which 47 lncRNAs and 20 miRNAs had upregulated expression and 36 lncRNAs and 15 miRNAs had downregulated expression in the samples from ASD patients and HVs, respectively (Figure 2).

### ceRNA network

**FIGURE 3:**
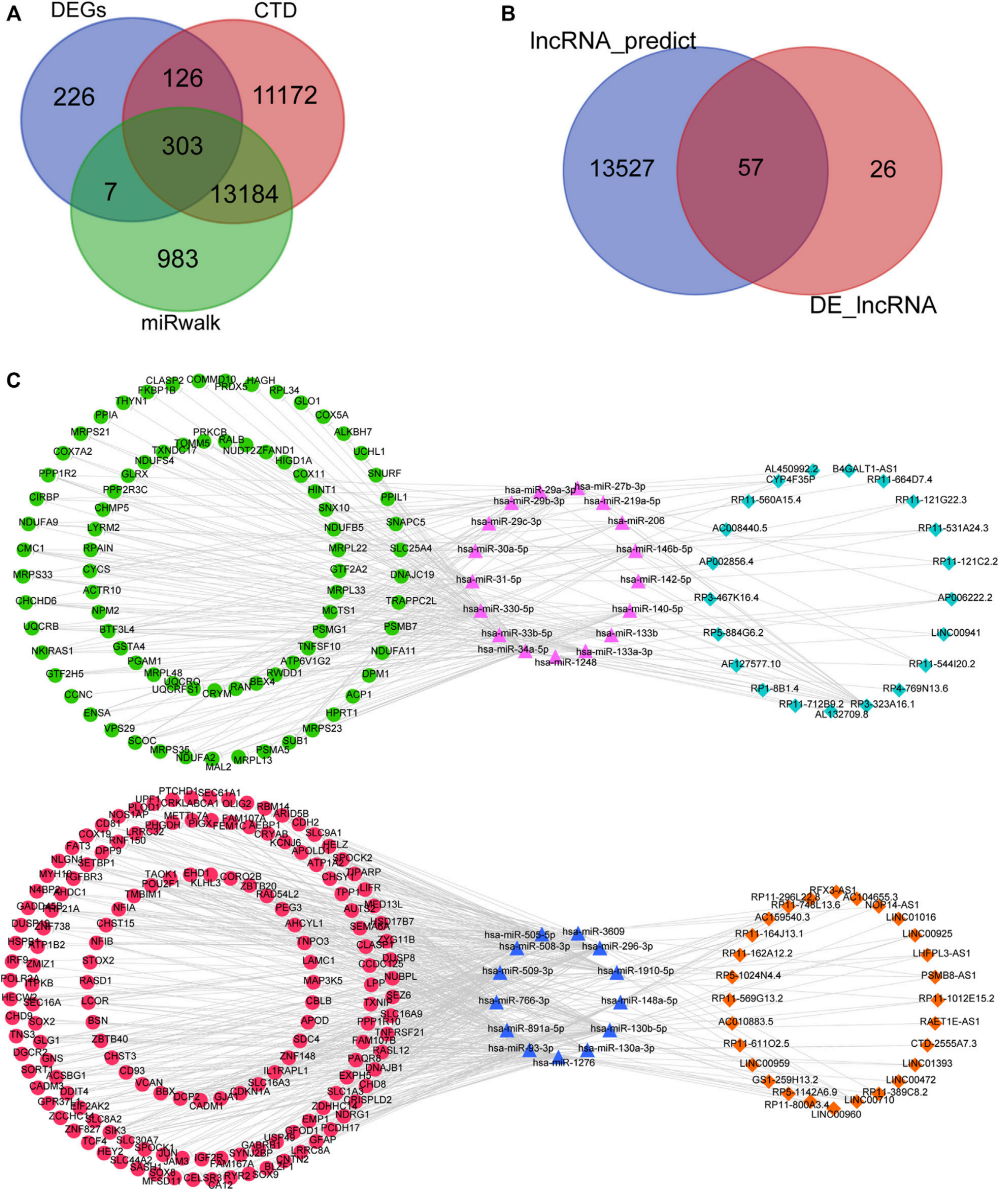
Construction of the ceRNA network. **(A)** Intersection of the differentially expressed mRNAs in the GSE38322 dataset, ASD-related mRNAs in the CTD, and the predicted mRNAs from differentially expressed miRNAs in the GSE59286 dataset. **(B)** Intersection of the differentially expressed lncRNAs in the GSE36315 dataset and lncRNAs predicted from differentially expressed miRNAs in the GSE59286 dataset. **(C)** lncRNA–miRNA–mRNA regulatory network constructed based on the mechanism of action of ceRNAs. Pink triangle: highly expressed miRNA; green dot: low expression of lncRNA; light-blue rhombus: low expression of mRNA; blue triangle: low expression of miRNA; red dot: high expression of lncRNA; orange rhombus: high expression of mRNA.

Based on the 35 differentially expressed miRNAs of the GSE59286 database, 14,477 mRNAs were predicted using the miRWalk database. By searching the CTD, 24,785 ASD-related mRNAs were obtained. Finally, 622 differentially expressed mRNAs were obtained from the GSE38322 dataset. We obtained 303 mRNAs after intersecting the three data sources mentioned above (Figure 3A). Furthermore, according to the reverse expression (one upregulated and the other downregulated) of miRNA and mRNA, 735 miRNA–mRNA pairs were obtained, which contained 35 miRNAs and 252 mRNAs.

Based on the 35 differentially expressed miRNAs of the GSE59286 database, 13,584 lncRNAs were predicted using the LncBaseV2 database. Subsequently, 83 lncRNAs were obtained from the differential-expression analysis of the GSE36315 dataset. Finally, 52 lncRNAs were obtained after intersecting the data from the lncBaseV2 database with analyses of differential expression (Figure 3B). According to the reverse expression (one upregulated and the other downregulated) of miRNA and lncRNA, 109 miRNA–lncRNA pairs were obtained, which contained 30 miRNAs and 49 lncRNAs. Moreover, among the 735 miRNA–mRNA pairs and 109 miRNA–lncRNA pairs obtained, the lncRNA–miRNA–mRNA associations with the same miRNA as the core were screened, and a ceRNA network consisting of 49 lncRNAs, 30 miRNAs, and 236 mRNAs was constructed (Figure 3C).

**FIGURE 4:**
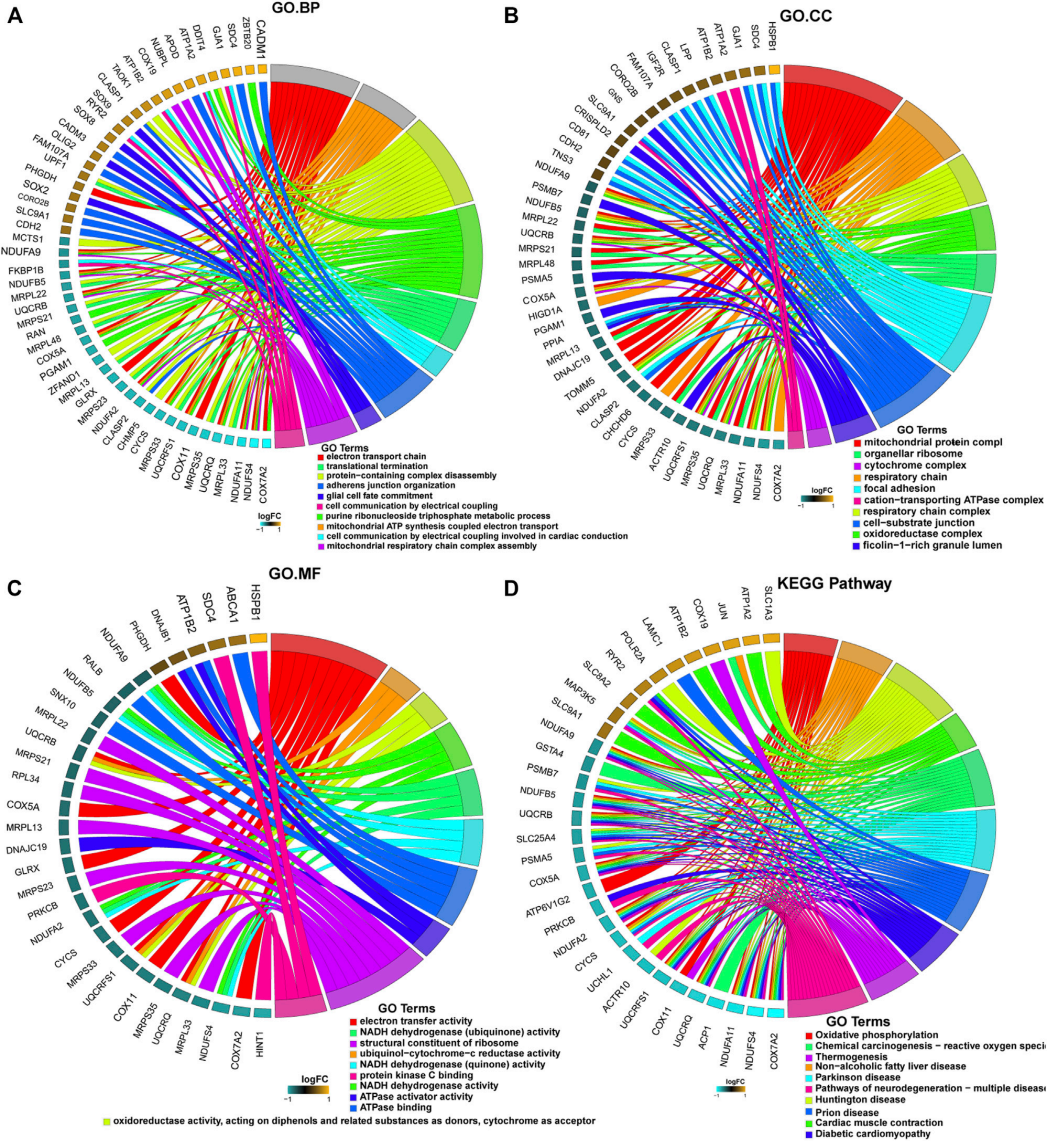
Enrichment analyses of mRNAs in the ceRNA network using the Gene Ontology (GO) database and Kyoto Encyclopedia of Genes and Genomes (KEGG) database. **(A)** Cellular components (CC); **(B)** biological process (BP); **(C)** molecular functions (MF); **(D)** regulatory signaling pathways.

Further enrichment analyses of the mRNAs in the ceRNA networks revealed that these genes were associated with 41 cellular components, including “mitochondrial protein complexes”, “organelle ribosomes”, “cytochrome complexes”, “respiratory chain”, “focal adhesion”, and “cation-transport ATPase complexes” (Figure 4A). These genes in the ceRNA network were also associated with 208 biological processes, including “electron transport chain”, “translation termination”, protein-containing complex disassembly”, “adhesion junction organization”, “glial cell fate commitment”, and “cell communication by electrical coupling” (Figure 4B); 39 molecular functions, including “electron transport activity”, “NADH dehydrogenase (ubiquinone) activity”, “structural components of ribosomes”, “ubiquinone-cytochrome c reductase activity”, “NADH dehydrogenase (quinone) activity”, and “protein kinase C binding” (Figure 4C). Finally, we found 35 regulatory signaling pathways associated with these genes, including “oxidative phosphorylation”, “reactive oxygen species”, and “multiple neurodegenerative disease pathways” such as Parkinson’s disease and Huntington’s disease (Figure 4D).

### Immune-cell infiltration in ASD patients

**FIGURE 5:**
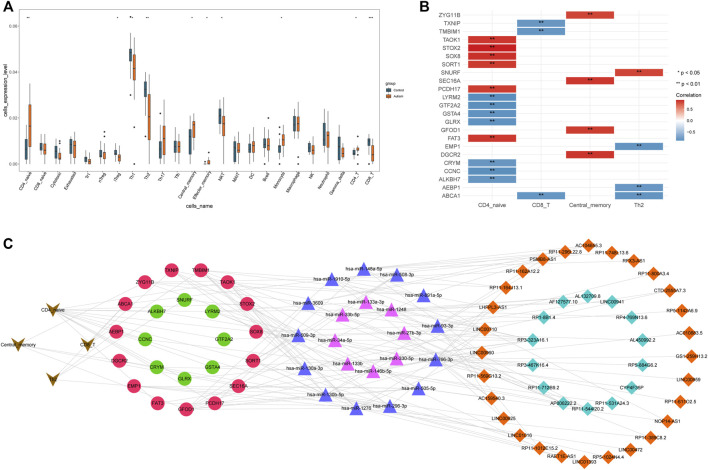
Construction of an immune cell-associated ceRNA network. **(A)** Infiltration of immune cells of ASD patients in the GSE38322 dataset; **(B)** immune cell–mRNA pairs; **(C)** Immune cell-associated ceRNA network.

Differential analysis of the infiltration of 24 immune-cell types in ASD patients and HVs showed that populations of cluster of differentiation (CD)4-initial cells (CD4^−^ naïve), induced regulatory T cells (iT_regs_), type-1 helper T cells (Th1), Th2 cells, central-memory cells, effector-memory cells, natural killer T (NKT) cells, monocytes, CD4^−^ T cells, CD8^−^ T cells, and 10 types of immune cells were significantly different between the two groups. Significantly more CD4^−^ naïve, central-memory, effector-memory, monocyte, and CD4^−^ T cells infiltrated in samples from ASD patients than in samples from HVs, whereas significantly fewer iT_regs_, Th1, Th2, NKT, and CD8^−^ T cells infiltrated in ASD patients than in HVs (Figure 5A).

Spearman correlation analysis of 10 types of immune cells and mRNAs with significantly different expression revealed 24 significantly correlated immune cell–mRNA pairs for four types of immune cells and 23 mRNAs (Figure 5B) at a threshold of *p* < 0.05 and |r| > 0.8. Subsequently, using Cytoscape, an immune cell-associated ceRNA network was constructed. This network generated 277 lncRNA–miRNA–mRNA–immune-cell associations from 42 lncRNAs, 21 miRNAs, 23 mRNAs, and four types of immune cells (Figure 5C).

### Construction of a diagnostic model

**FIGURE 6:**
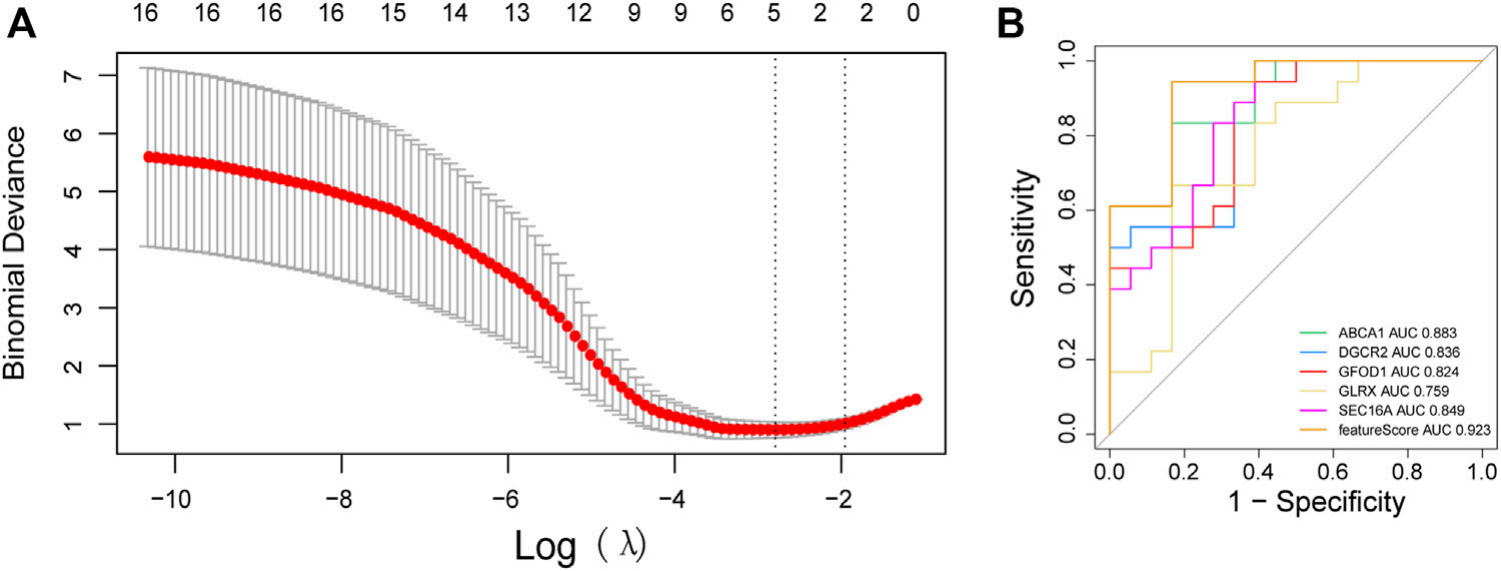
Construction of a model for genetic diagnostic markers. **(A)** *λ* selection plot in the LASSO model; **(B)** ROC curve of the diagnostic model and diagnostic markers.

We undertook LASSO regression analyses on 23 mRNAs associated with immune cells under 20-fold cross-validation (Figure 6A) and screened five ASD-associated diagnostic marker genes: adenosine triphosphate-binding cassette transporter A1 (*ABCA1*), DiGeorge syndrome critical region 2 (*DGCR2*), glucose-fructose oxidoreductase structural domain gene 1 (*GFOD1*), glutaredoxin (*GLRX*), and SEC16 homolog A (*SEC16A*). Based on the regression coefficient of each gene, the sample eigenvalue was calculated as:

Feature = ABCA1ch × −1.719843535 + DGCR2 × −0.003835302 + GFOD1 × −0.277741202 + GLRX × 0.01064725 + SEC16A × −1.697826627.

Analyses of the ROC curve of the model composed of the five diagnostic genes revealed that the area under the ROC curve (AUC) of the model was ≤0.923: the diagnostic performance of the model was good. Single-gene analyses of the ROC curve revealed an AUC for *ABCA1* of 0.883, whereas for *DGCR2* it was 0.836, for *GFOD1* it was 0.824, for *GLRX* it was 0.759, and for *SEC16A* it was 0.849. Hence, single genes could also be used as diagnostic biomarkers (Figure 6B).

### GSEA of each diagnostic marker gene-associated gene set

**FIGURE 7:**
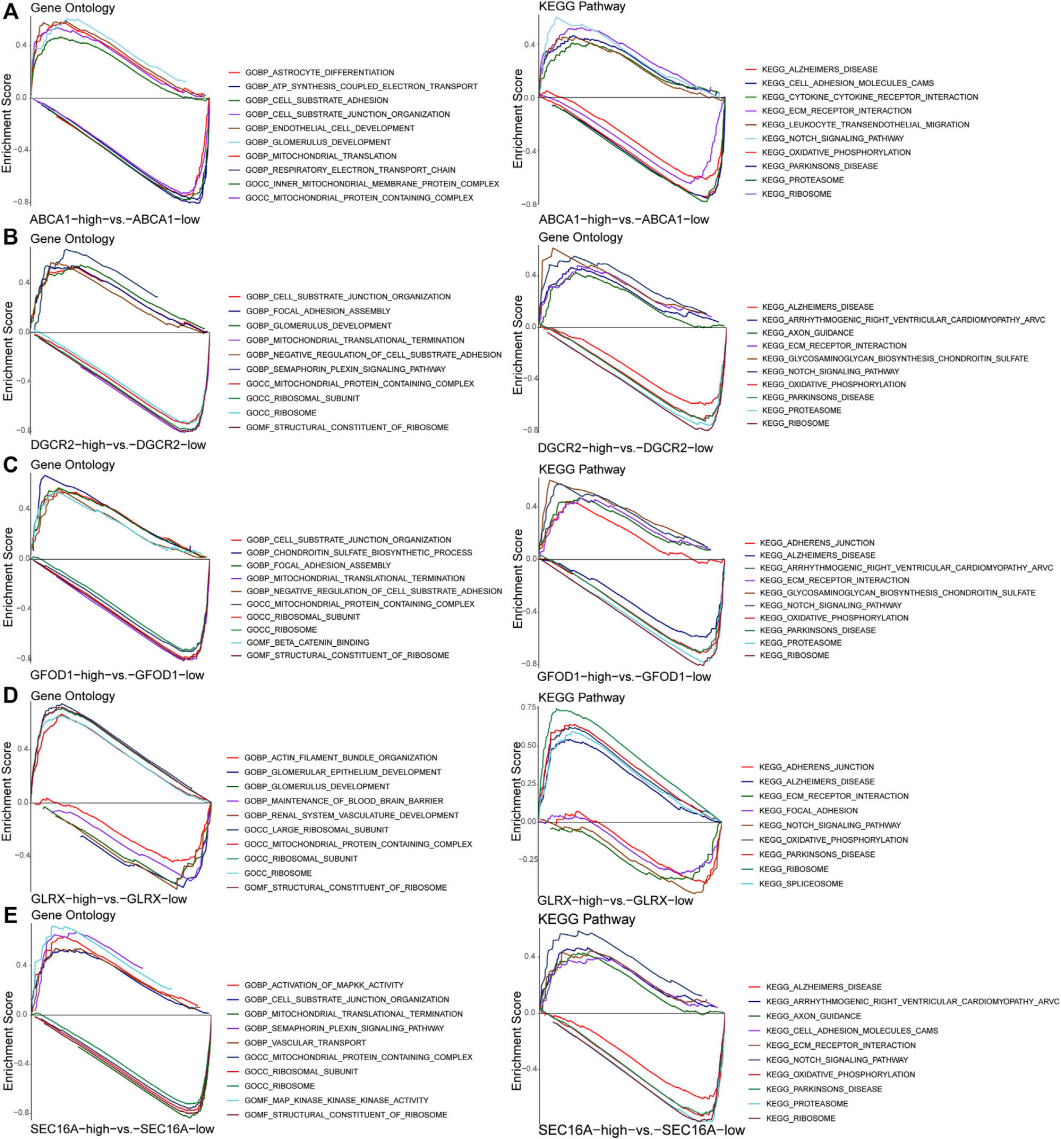
Gene set enrichment analysis of the diagnostic marker genes *ABCA1* **(A)**, *DGCR2* **(B)**, *GFOD1* **(C)**, *GLRX* **(D)**, and *SEC16A* **(E)**.

After GSEA for screened ASD-associated diagnostic marker genes, the top-10 pathways (according to GO and KEGG databases) for each diagnostic marker gene ranked by FDR q-values were obtained (Figures 7A–E). These results suggested that marker genes-associated gene sets were enriched mainly in immune-response signaling pathways.

### Validation of expression of diagnostic marker genes

**FIGURE 8:**
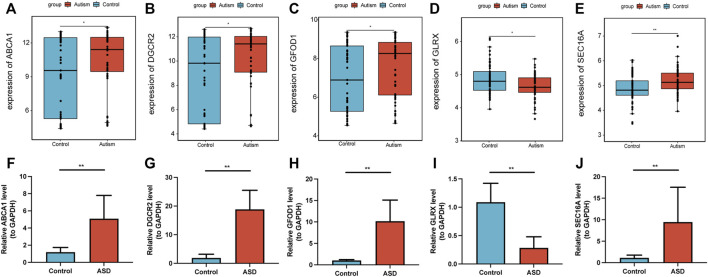
Validation of expression of diagnostic marker genes. Expression of the diagnostic marker genes *ABCA1* **(A)**, *DGCR2* **(B)**, *GFOD1* **(C)**, *GLRX* **(D)**, and *SEC16A* **(E)** in the GSE28475 dataset. Expression of the diagnostic marker genes *ABCA1* **(F)**, *DGCR2* **(G)**, *GFOD1* **(H)**, *GLRX* **(I)**, and *SEC16A* **(J)** in serum samples. *p < 0.05, **p < 0.01.

mRNA expression of the five diagnostic marker genes for ASD (*ABCA1, DGCR2, GFOD1, GLRX*, and *SEC16A*) was analyzed in the GSE28475 dataset and the serum samples collected from study participants (Figure 8). Expression of *ABCA1, DGCR2, GFOD1*, and *SEC16A* was significantly higher, whereas that of *GLRX* was lower, in ASD patients than that in HVs. Hence, these five genes showed differential expression in ASD patients, which was consistent with the diagnostic model.

## Discussion

Since the first report of autism in 1943 by Kanner, understanding of this disease has changed dramatically. Initially, ASD was thought to be caused by inappropriate parenting practices. However, it was discovered later that ASD is a developmental disorder of the central nervous system (CNS), and individuals who carry rare *de novo* genetic mutations are at a greater risk of developing ASD. Furthermore, it was not until the 1980s that, due to the development of epigenetics, ASD was recognized to be the result of a combination of environmental stimuli in a certain genetic background.

Sequencing has been the most promising strategy to reveal the pathogenesis and diagnosis of ASD (Stefanski et al., 2021). Through an exome-sequencing study of >10,000 ASD patients, Satterstrom et al. found most ASD-associated genes to show differential expression in the early stages of brain development, and that these genes primarily regulated gene transcription, neuronal development, and physiological function (Satterstrom et al., 2020). By taking a single-cell genomics approach, Velmeshev et al. found that differentially expressed genes in ASD patients were concentrated in excitatory neurons and microglia in the cerebral cortex, and were functionally enriched in neuronal projections and adhesions. Furthermore, differential expression of specific genes in cortical–cortical projection neurons correlated with the severity of the clinical manifestations of ASD (Velmeshev et al., 2019). Through whole-exome sequencing, Liu et al. found that the function of the genes associated with gut microbes in ASD patients was mainly enriched in the innate immune response, protein glycosylation, and retrograde axonal transport, which emphasized the role of the brain–gut axis and neuroimmunity in ASD development (Liu et al., 2021). However, even though >1,000 mutations, chromosomal abnormalities, and copy-number variants have been associated with ASD development, each type of variant cannot be used to explain most cases due to the heterogeneity of ASD.

An approach using bioinformatics analysis can be helpful in this scenario. A weighted gene co-expression network was constructed to analyze the genes known to be related to ASD, and to find intersections of these risk genes to discover novel diagnostic and therapeutic options (Parikshak et al., 2015). We attempted to obtain an ASD-related ceRNA regulatory network through bioinformatics analysis. Furthermore, we combined our ceRNA network with differential analyses of immune-cell infiltration to identify ASD biomarkers.

miRNAs are at the core of a ceRNA network. There have been many reports of abnormal expression of multiple miRNAs in the brain tissue and peripheral blood of ASD patients (Xiong et al., 2020). Given that one miRNA can simultaneously regulate expression of multiple genes, and that each gene is also regulated by multiple miRNAs, an “miRNA imbalance” may explain the heterogeneity among ASD patients. Moreover, miRNAs are epigenetic regulatory molecules, so changes in their expression can help unravel the link between heredity and the environment (Masini et al., 2020).

We constructed a ceRNA network comprising 49 lncRNAs, 30 miRNAs, and 236 mRNAs by processing the differentially expressed miRNAs, mRNAs, and lncRNAs obtained from targeting predictions in databases. Through analyses of functional enrichment, we discovered that the top-10 biological processes and molecular functions involved in the genes in this network were mainly related to mitochondrial-energy metabolism, cellular communication, transcriptional regulation, and glial-cell fate, whereas the main signaling pathways involved were associated with energy metabolism and neurodegeneration. These findings are consistent with the underlying pathological changes in ASD: examinations using functional magnetic resonance have demonstrated abnormal activation/inhibition of several brain regions involved in social interaction and information integration in the brains of ASD patients. Moreover, neuropathological examinations have revealed extensive abnormalities in synaptic development/structure and brain function in ASD patients. The brain is a region of high energy metabolism whose neurons are highly dependent on mitochondrial energy metabolism to support neurogenesis, signaling, and synaptic remodeling (Hollis et al., 2017). Furthermore, transcriptional regulation and glial-cell fate can influence neuronal activity and synaptic development, while themselves being highly dependent on mitochondrial energy supply. Hence, mitochondrial dysfunction and abnormal energy metabolism may be a potential common mechanism in ASD pathogenesis.

Studies have demonstrated immune dysregulation, inflammation, and endogenous production of autoantibodies due to abnormalities of innate and adaptive immune systems in ASD patients (Meltzer and Van de Water, 2017). Autoantibodies against neuronal antigens have been detected in children with ASD and their mothers. Moreover, use of immunoglobulin-G from the mothers of mice and rhesus monkeys with ASD has been shown to induce ASD-like symptoms in their offspring. Those findings suggest that the humoral immune system is critical for ASD pathogenesis (Gupta et al., 2010).

We discovered infiltration of fewer iT_regs_ as well as Th1, Th2, NKT, and CD8^−^ T cells in ASD patients than in HVs. Infiltration of more CD4−initial, central-memory, effector-memory, monocytes, and CD4^−^ T cells was noted, which suggested that cellular immunity was also associated with ASD development. Mostafa et al. showed a reduction in the number of CD4^+^CD25^high^ T_regs_ in ASD children (Mostafa et al., 2010). Abdallah et al. reported a reduction in cytokine secretion by Th1 and Th2 cells in children with ASD (Abdallah et al., 2012). Enstrom et al. found a lower responsiveness by NK cells in children with ASD (Enstrom et al., 2009) and an increase in monocyte response (Enstrom et al., 2010). Interestingly, some scholars have reached different conclusions. Gupta et al. reported a reduction in the number of CD8^+^CCR7^+^ CD45RA CD8^+^ central-memory T cells in ASD children (Gupta et al., 2010). Ashwood et al. suggested that increased responses from Th1 cells were associated with the core symptoms of ASD (Ashwood et al., 2011). We noted increased infiltration of Th17 cells (which have been reported to be associated with ASD (Choi et al., 2016)) but the difference was not significant. Thus, even though a heterogeneous pattern of changes in the immune cells of patients with ASD is apparent (which may be related to differences in samples and selection of different HVs), there is little doubt that cellular immunity participates in ASD development, and that clinical application of immunomodulators (e.g., pro-immunoglobulin and IL-6 antagonists) improves (at least in part) ASD symptoms in children.

Given the relevance of the immune system in ASD, we used an established ceRNA network to synthesize the results of an analysis of immune cell infiltration, and we obtained five diagnostic genes using the LASSO algorithm: *ABCA1, DGCR2, GFOD1, GLRX*, and *SEC16A*. Analyses of ROC curves revealed that the diagnostic model based on a combination of these five diagnostic genes had an AUC of 0.923. This value is higher than the AUC (0.910) of the three-methylation-markers diagnostic model identified using the same algorithm (Zhang et al., 2022), the AUC (0.910) of the five-gut-bacterial-markers diagnostic model identified by a metagenomic analysis combined with a random forest algorithm (Wan et al., 2022), and the AUC (0.860) of the autism-risk-index diagnostic method based on eye-tracking measures (Frazier et al., 2018), but slightly lower than the AUC (0.947) of the DarkASDNet diagnostic model based on 3D-fMRI (Ahammed et al., 2021). Finally, we confirmed the expression of these five genes in collected serum samples and the validation dataset. The results showed that the five genes had differential expression in ASD patients, which was consistent with the trend predicted by the diagnostic model.

ABCA1 is a transmembrane protein that maintains cellular cholesterol homeostasis and affects the transport of free cholesterol and phospholipids (Brousseau, 2003). Within the nervous system, ABCA1 is expressed mainly in neurons and astrocytes. ABCA1 is associated with apolipoprotein 1-mediated transport of cholesterol and phospholipids, which is involved in the development of Alzheimer’s disease and Parkinson’s disease. ABCA1 is also associated with cardiolipin-driven mitochondrial dysfunction (Jacobo-Albavera et al., 2021). Approximately 23% of the body’s cholesterol is found in the CNS, and it is an important raw material for the growth and differentiation of neurons. Moreover, metabolic dysregulation characterized by reduced phospholipid levels in the brain and plasma has been implicated in ASD pathogenesis (Alfawaz et al., 2018).

*DGCR2* is associated with susceptibility to schizophrenia, and is expressed throughout brain development. *DGCR2* encodes an activation-dependent adhesion protein involved in regulating the migration and localization of neurons during early corticogenesis (Molinard-Chenu and Dayer, 2018). Belangero et al. found that certain mutations in *DGCR2* may also be associated with cortical thickness (Belangero et al., 2019). Abnormal projections of cortex–cortical circuits (Chow et al., 2011) and abnormal cortical growth during infancy (Hazlett et al., 2017) are also present in the brains of ASD patients, which suggests that DGCR2 may be involved in ASD development. However, the exact mechanism must be investigated.

*GLRX* encodes GLRX, a crucial member of the dithiol-disulfide oxidoreductase family, which is involved in regulation of the transcription factors involved in antioxidative stress and synthesis of control DNA by regulating the activity of ribonucleotide reductase (Chang et al., 2020). Studies have demonstrated increased oxidative stress in the brain of children with ASD, increased levels of glutathione disulfide, and decreased levels of glutathione/glutathione disulfide (reduced/oxidized glutathione) in peripheral blood (Chen et al., 2021). Bowers and colleagues found, through a family-lineage study, that mutations in *GLRX* (a critical component of the glutathione-GLRX reduction system) resulted in a fourfold increase in ASD risk (Bowers et al., 2011).

Little research has been done on *GFOD1*, but it shows high expression in the early development of zebrafish brains. *GFOD1* may be associated with neurons that produce gamma-aminobutyric acid (Lechermeier et al., 2020). Increased expression of *GFOD1* has been noted in the hippocampal tissue of patients with temporal-lobe epilepsy and patients with attention deficit hyperactivity disorder (Lasky-Su et al., 2008; Dixit et al., 2016). Trent et al. found increased expression of GFOD1 in a study of X^Y*^O mice (a genetically defective mouse associated with attention deficit hyperactivity disorder and ASD) (Trent et al., 2014), but the exact function of the GFOD1 protein is not known.

*SEC16A* encodes an endoplasmic-reticulum exit-site scaffolding protein, which is an important component of coat protein complex II (COP II) vesicles and mediates the transport of substances from the endoplasmic reticulum to the Golgi apparatus (Hughes and Stephens, 2010). COPII is involved in metabotropic glutamate-mediated long-range inhibition (Pick et al., 2017), and defective long-term depression of metabotropic glutamate receptors is associated with ASD development (Li et al., 2015). COPII is also involved in the development of Crohn’s disease and obligatory spondylitis (Nakamura et al., 2021). Hence, *SEC16A* may be involved in ASD development through the immune system.

Rapid advancement of sequencing technology has led to great progress in screening for the diagnostic markers of ASD, but it had two main limitations in our study. First, the sequencing results for mRNAs, lncRNAs, and miRNAs arose from three datasets and belonged to different patients, which brings uncertainty to the diagnosis of ASD. Second, the results of bioinformatics analysis must be validated by clinical and *in vitro* studies, but the sample size of our validation dataset was small.

## Conclusion

We constructed an ASD-associated ceRNA regulatory network. This network will provide a new perspective for explaining the biological processes of ASD. Furthermore, we identified five immune cell-associated potential molecular marker genes of ASD, which could be novel targets for therapy and immunotherapy strategies against ASD.

## Data Availability

The datasets presented in this study can be found in online repositories. The names of the repository/repositories and accession number(s) can be found in the article/Supplementary Material.
